# Serum pharmacodynamic biomarkers for chronic corticosteroid treatment of children

**DOI:** 10.1038/srep31727

**Published:** 2016-08-17

**Authors:** Yetrib Hathout, Laurie S. Conklin, Haeri Seol, Heather Gordish-Dressman, Kristy J. Brown, Lauren P. Morgenroth, Kanneboyina Nagaraju, Christopher R. Heier, Jesse M. Damsker, John N. van den Anker, Erik Henricson, Paula R. Clemens, Jean K. Mah, Craig McDonald, Eric P. Hoffman

**Affiliations:** 1 Research Center for Genetic Medicine, Children’s National Health Systems, Washington, DC 20010, USA; 2 Department of Physical Medicine & Rehabilitation, University of California, Davis School of Medicine, Davis, CA 95618, USA; 3 Neurology Service, Department of Veterans Affairs Medical Center, Department of Neurology, University of Pittsburgh, Pittsburgh, PA, USA; 4 Department of Pediatrics, Alberta Children’s Hospital, Calgary, AB, T3B 6A8 Canada

## Abstract

Corticosteroids are extensively used in pediatrics, yet the burden of side effects is significant. Availability of a simple, fast, and reliable biochemical read out of steroidal drug pharmacodynamics could enable a rapid and objective assessment of safety and efficacy of corticosteroids and aid development of corticosteroid replacement drugs. To identify potential corticosteroid responsive biomarkers we performed proteome profiling of serum samples from DMD and IBD patients with and without corticosteroid treatment using SOMAscan aptamer panel testing 1,129 proteins in <0.1 cc of sera. Ten pro-inflammatory proteins were elevated in untreated patients and suppressed by corticosteroids (MMP12, IL22RA2, CCL22, IGFBP2, FCER2, LY9, ITGa1/b1, LTa1/b2, ANGPT2 and FGG). These are candidate biomarkers for anti-inflammatory efficacy of corticosteroids. Known safety concerns were validated, including elevated non-fasting insulin (insulin resistance), and elevated angiotensinogen (salt retention). These were extended by new candidates for metabolism disturbances (leptin, afamin), stunting of growth (growth hormone binding protein), and connective tissue remodeling (MMP3). Significant suppression of multiple adrenal steroid hormones was also seen in treated children (reductions of 17-hydroxyprogesterone, corticosterone, 11-deoxycortisol and testosterone). A panel of new pharmacodynamic biomarkers for corticosteroids in children was defined. Future studies will need to bridge specific biomarkers to mechanism of drug action, and specific clinical outcomes.

Corticosteroids have been prescribed for a broad array of adult and pediatric inflammatory conditions since the 1950’s, with approximately 2–3% of the general population prescribed glucocorticoids on an annualized basis^1^,^2^. Chronic use is associated with multiple side effects, where the severities of the safety concerns vary with dose, dose duration, and age^3^,^4^. For example, corticosteroids cause changes in bone remodeling leading to stunting of growth and vertebral fractures in children^5^, and exacerbation of osteopenia and fracture in the elderly^6^. Additional side effects that can alter quality of life of the child and family include weight gain, Cushingoid appearance, mood changes, sleep disturbances, and immune suppression. Daily high dose corticosteroids are considered standard of care for Duchenne muscular dystrophy, but the balance of efficacy (greater strength and later loss of ambulation), are offset to a variable degree by side effects, leading to wide variations in practice and discontinuation of treatment^7^,^8^. Similarly, corticosteroids are effective for children with inflammatory bowel disease, but metabolic side effects may exacerbate nutritional concerns, and there is a movement towards use of biologics to avoid the side effects of corticosteroids^9^. Efforts in finding a better balance between efficacy and safety have included the evaluation of alternative dosing schedules and use of different types or formulations of corticosteroids^10^,^11^. Also, drug development programs aiming at dissociation of efficacy from side effects are emerging^12^.

Robust pharmacodynamic biomarkers for corticosteroids bridged to clinical outcomes could serve as acute, objective read outs for aspects of both efficacy and safety. Some well-established safety concerns of corticosteroids have existing biomarkers well-bridged to clinical outcomes, namely adrenal suppression, insulin resistance, bone turnover, and immune suppression^13^. Corticosteroids suppress ACTH secretion by the pituitary, and adrenal production of cortisol within hours of the first dose. With chronic dosing, persistent suppression of adrenal function can lead to adrenal atrophy, and adrenal insufficiency where the adrenal cortex is unable to respond to increased circulating ACTH. Adrenal insufficiency in turn leads to a lack of ability to mount a cortisol response in times of acute stress (trauma, surgery, acute illness), and thus increased risk for potentially lethal adrenal crisis (hypotension, hypoglycemia)^3^,^14^. Measure of first-in-morning serum cortisol >24 hours after the last corticosteroid dose is a reliable measure of adrenal suppression, but is non-specific for adrenal insufficiency and risk for adrenal crisis. Corticosteroids induce both acute and chronic insulin resistance, and this is measured by fasting glucose and insulin levels^15^. Corticosteroids also cause acute and chronic decreases in bone turnover markers (osteocalcin, procollagen 1-N and C-terminal peptides), and these have been utilized as pharmacodynamic safety biomarkers for osteopenia in clinical trials of osteoarthritis and other disorders^16^. Immune suppression can be monitored by circulating levels of lymphocytes, and corticosteroid-induced reductions are seen both acutely and chronically^13^. To our knowledge, no broad discovery program has been carried out to define novel safety and efficacy biomarkers, in either adults or children.

Emerging technologies permit a broad-based analysis of proteins in small quantities of serum (proteomics). One particularly robust emerging proteomics platform is the SOMAscan technology, where specific aptamers (small protein-binding fragments of nucleic acid) have been developed as sensitive and reliable tests for 1,129 serum proteins measured simultaneously in <0.1 cc of serum^17^,^18^. Here, we applied this technology to identify corticosteroid-responsive pharmacodynamic biomarkers in children with Duchenne muscular dystrophy and inflammatory bowel disease.

## Results

### Corticosteroid-responsive serum biomarker discovery using a cross-sectional group of steroid-naïve and steroid-treated DMD patients

An age-adjusted comparison of corticosteroid treated to corticosteroid naïve DMD patients identified significant alterations in the levels of 22 proteins; 8 elevated and 14 decreased by drug (Table 1). Concentrations of the steroid-responsive proteins were then compared to untreated age-matched healthy boys. Three proteins insulin (INS), soluble form of growth hormone receptor (GHBP) and leptin (LEP) that did not meet statistical significance in the cross-sectional data, but were found to be significant in one or both longitudinal sample sets of pre and post corticosteroid treated DMD or IBD patients were retained for analysis.

**Figure 1:**
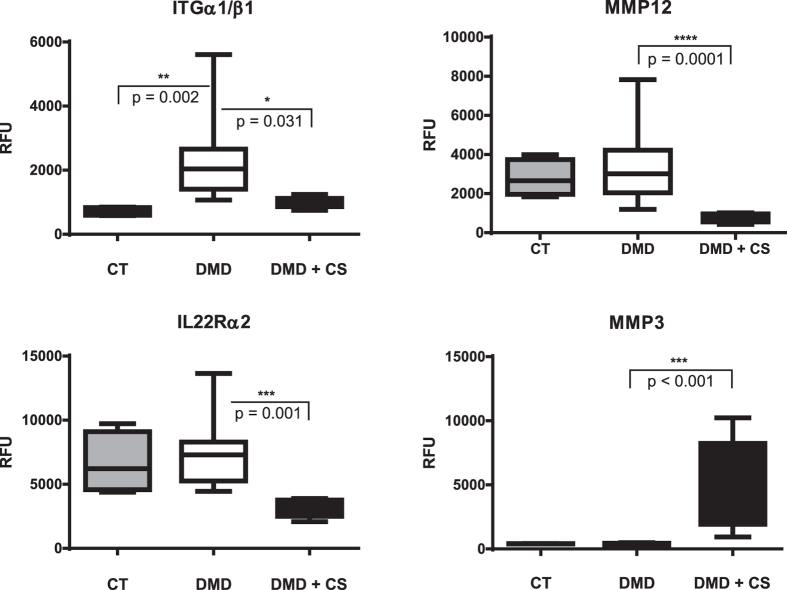
Corticosteroid-responsive serum protein biomarkers in children with DMD. Shown are SOMAscan analyses of cross-sectional groups of corticosteroid-naïve children (DMD; n = 9), corticosteroid-treated children (DMD + CS; n = 5), and healthy children (CT; n = 4). ITGa1/b1: Integrin alpha-I/beta-1 complex. IL22RA2: interleukin-22 receptor subunit alpha2; MMP12: matrix metalloproteinase 12 also known as macrophage metalloelastase; MMP3: matrix metalloproteinase 3 also known as stromelysin 1. Values for each protein are plotted as RFU (relative fluorescent units) and represent the average mean and standard error in each group.

The 22 identified steroid-responsive proteins can be classified into 3 groups. The first group comprised 5 biomarkers that were significantly increased in the steroid-naïve DMD relative to untreated age matched healthy controls but decreased in corticosteroid-treated DMD toward the concentrations seen in the control group. These biomarkers were generally inflammatory proteins, reflective of the chronic pro-inflammatory state in DMD patients and were suppressed by corticosteroid treatment, and thus could be considered potential corticosteroid anti-inflammatory efficacy biomarkers. These included chemokine (C-C) motif chemokine 22 (CCL22) also known as macrophage-derived chemokine (MDC), insulin-like growth factor binding protein 2 (IGFBP-2), complement receptor type 1 (C1R), integrin alpha-I/beta-1 complex (ITGa1/b1) and fibrinogen gamma (FGG). A representative exemplar of this group is shown for ITGa1/b1 in Fig. 1.

The second group of corticosteroid-responsive biomarkers consisted of nine proteins that were significantly reduced in their concentrations in the drug-treated DMD group relative to steroid-naïve DMD group but did not differ in their concentrations between steroid-naïve DMD group and untreated controls. These included macrophage metalloelastase (MMP12), interleukin-22 receptor subunit alpha2 (IL22Rα2) also known as IL-22BP, low affinity immunoglobulin epsilon Fc receptor (FCER2) also known as CD23, chemokine ligand 6Ckine CCL21 21(CCL21), T-lymphocyte surface antigen Ly-9/CD229 (LY9), SLIT/NTRK-like protein 5 (SLITRK5), lymphotoxin alpha1/beta2 (LTa1/b2), angiopoietin-2 (ANGPT2) and the macrophage colony-stimulating factor 1 receptor (CSF1R). All these protein are associated with macrophage and T-lymphocytes and might reflect modulation of immune system by corticosteroid. Figure 1 shows representative exemplars for IL22Rα2 and MMP12.

Finally, the third group of corticosteroid-responsive biomarkers comprised five proteins that were similar in steroid-naïve DMD patients versus untreated controls, but were significantly increased by corticosteroid treatment in DMD. This third group included matrix metalloproteinase 3 (MMP3) also known as stromelysin 1, carnosine dipeptidase 1 (CNDP1), afamin (AFM), angiotensinogen (AGT), protein C (PROC), insulin (INS), leptin (LEP) and GHBP and were enriched for known safety issues with corticosteroids reflective of metabolism disturbances, with the exception of PROC which reflects an anti-inflammatory effect of corticosteroid as discussed below. A representative exemplar of these corticosteroid safety biomarkers is shown for MMP3 (Fig. 1).

### Confirmation of corticosteroid-responsive biomarkers using longitudinal serum samples from pre- and post-corticosteroid-treated patients

Corticosteroid responsiveness of the biomarkers was tested in two independent longitudinal sample sets: serum collected from pre- and post-corticosteroid treated DMD patients and from pre- and post-corticosteroid treated IBD patients. Since these samples were collected through natural history studies, patients often had variable corticosteroid regimens, treatment durations and multiple sampling time points (Supplemental Tables S1 and S2). All pre- and post-treatment data points were used in the analysis while adjusting for both corticosteroid use and treatment duration (Table 2).

Of the 22 corticosteroid-responsive cross-sectional biomarkers (Table 1), 17 were confirmed by one or both of the longitudinal analyses (Table 2). MMP12, ITGa1/b, LTa1/b2, LEP and PROC were significantly altered in pre- and post-corticosteroid-treated DMD but not in pre- and post-corticosteroid-treated IBD. These differences could be attributed to the short term corticosteroid treatment in IBD (average 2 months) versus the long term treatment in DMD (average 4 months).

### Validation of SOMAscan data using ELISA assays

**Figure 2:**
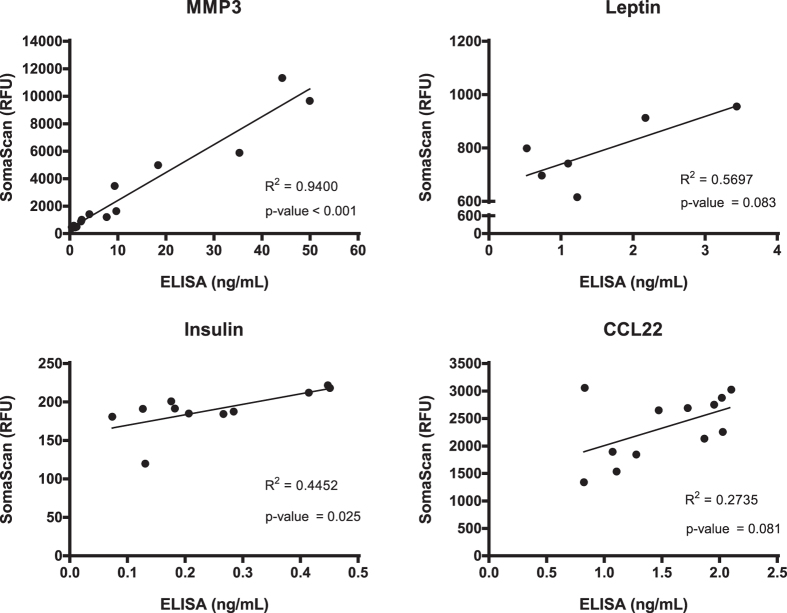
Correlation of SOMAscan and ELISA assays for corticosteroid-responsive proteins. Serum samples from DMD boys were tested in parallel by the two methods, and values plotted. The number of samples tested was different from protein to protein due the availability of serum samples.

Four GC-responsive protein biomarkers identified by SOMAscan were validated using a non-array method (ELISA assays) (CCL22, MMP3, insulin and leptin; (Fig. 2). The ELISA data closely agreed with SOMAscan data in each of the four proteins tested. There was evidence of a positive relationship between ELISA and SOMAscan data in each of the four proteins tested. This relationship was statistically significant at the p < = 0.05 level for MMP 3 (r2 = 0.94, p-value < 0.001) and insulin (r2 = 0.45, p-value = 0.025). The relationships for Leptin (r2 = 0.57, p-value = 0.083) and CCL22 (r2 = 0.27, p-value = 0.081), while positive and in similar direction of change, did not reach statistical significance.

### Corticosteroid treatment causes broad reductions in adrenal steroid hormones in DMD and IBD patients

**Figure 3:**
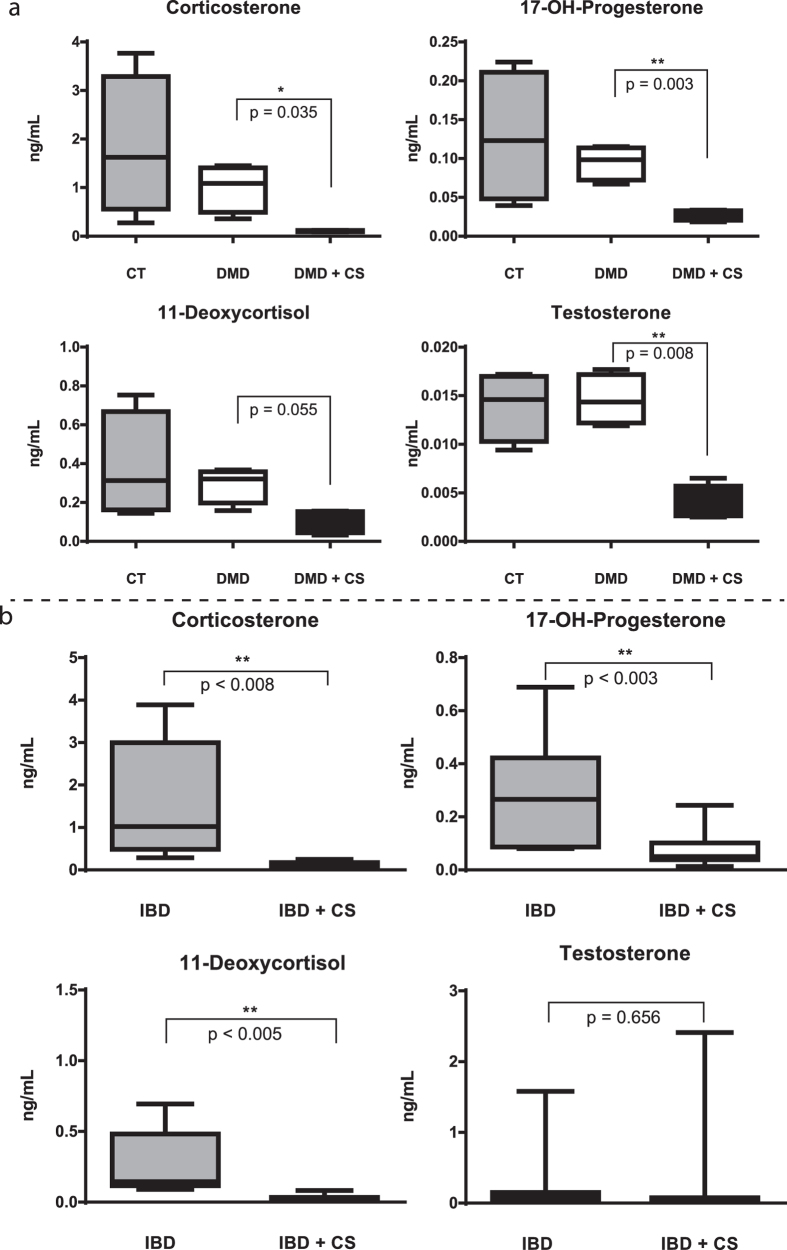
Adrenal steroidal hormones are suppressed by chronic corticosteroid treatment in DMD and IBD children. (**a**) Longitudinal analysis of DMD children (n = 9) and age-matched controls (n = 4). (**b**) Longitudinal analysis of IBD children (n = 11). Values for steroids are plotted in ng/mL and represent the average mean and standard error in each group.

A well-known safety concern with pharmacological corticosteroids is suppression of the adrenal cortex, with loss of diurnal cortisol production, and possible adrenal insufficiency. To our knowledge, other adrenal steroid hormones have not been well studied. Serum samples from pre and post-corticosteroid treated DMD and IBD patients were tested for levels of additional adrenal steroids using a mass spectrometry-based assay with stable isotope standards. All four steroid hormones tested were seen to be strongly reduced by corticosteroid treatment in DMD patients: 17-hydroxy-progesterone, corticosterone, 11-deoxycortisol and testosterone (Fig. 3a). This same panel of steroid hormones, except for testosterone, was also significantly decreased in IBD patients after corticosteroid treatment (Fig. 3b). Testosterone showed a very high degree of variation in IBD subjects and did not show significant steroid effect. This could be due to the inclusion of both boys and girls in the IBD group, and the shorter duration of corticosteroid treatment. This hypothesis is in agreement with the effect of corticosteroid on testosterone levels in respiratory disease patients^19^.

## Discussion

Using a combination of a highly multiplexed proteome profiling strategy and a steroid hormone profiling method we identified a panel of 21 serum circulating biomarkers (17 proteins and 4 steroid hormones) that were responsive to chronic daily corticosteroid treatment in children. The panel was defined through the study of three independent patient groups (cross-sectional DMD, longitudinal DMD, longitudinal IBD), with comparison to age matched healthy children.

Circulating levels of 17-hydroxyprogesterone, corticosterone, and 11-deoxycortisol were strongly reduced by chronic corticosteroid treatment in longitudinal studies of both DMD and IBD. Endogenous cortisol and pharmacological corticosteroids are known to repress the secretion of CRH (corticotrophin releasing hormone) from the hypothalamus, and in turn reduce secretion of ACTH (adrenocorticotropic hormone) from the pituitary, leading to less stimulation of the adrenal cortex, and less production of adrenal steroids (HPA axis; hippocampus-pituitary-adrenal axis). However, circulating levels of the axis is under strong diurnal control, and also strong regulation by stressors. Thus, there tend to be wide variations in levels both within and between subjects. To our knowledge, our study is the first to show broad steady state depression of 4 adrenal steroid hormones (e.g. 17-hydroxy-progesterone, corticosterone, 11-deoxycortisol and testosterone) in corticosteroid-treated children to levels well below the lower limit of normal age-matched controls. Adrenal insufficiency is commonly discussed in terms of failure to mount a stress response, and risk for adrenal crisis. However, this data shows that adrenal insufficiency has broader impacts on global adrenal steroids, likely explaining the observed delay of puberty in corticosteroid-treated DMD boys, and other endocrine disturbances^20^.

SOMAscan proteome profiling identified both known and novel corticosteroid-responsive serum protein biomarkers. The serum proteins that decreased in response to corticosteroid treatment were associated with inflammation and T-lymphocyte modulation reflecting the anti-inflammatory effects of corticosteroid and thus could be considered as corticosteroid efficacy biomarkers. MMP12 (macrophage activity), CCL22 (eosinophil trafficking), IL22RA2 (soluble receptor of IL-22), FCER2 (B cell function) and LY9 (T cell activation) are produced by different subtypes of macrophages and dendritic cells and modulate the immune and inflammatory system via maturation and activation of macrophages, T cells and B cells^21^,^22^,^23^,^24^,^25^,^26^. A common biochemical theme shared by these inflammatory proteins is their involvement in NF-kappa B pathways (danger signals of innate immunity), where NF-kappa B receptor complexes are inhibited by ligand-activated glucocorticoid receptor^27^.

Among these potential corticosteroid efficacy biomarkers, some have been previously reported showing steroid-responsive decreases in animal model systems or other adult inflammatory disorders. MMP12 was reported to be decreased by dexamethasone in an asthmatic mouse model^28^, CCL22 by dexamethasone in patients with atopic dermatitis^22^, FCER2 by prednisone in patients with giant cell arteritis^29^, LTa1/b2 by prednisone in patients with acquired hemophilia^30^, and IGFBP2 by deflazacort after renal transplantation^31^. The corticosteroid-responsive decreases in serum concentrations of the remaining markers IL22RA2, LY9, ITGa1/b1, ANGPT2 and FGG by corticosteroids are novel to our report.

In addition to the pro-inflammatory markers in DMD sera suppressed by corticosteroids (candidate efficacy markers), there were also proteins that showed similar levels comparing DMD to age-matched controls that were then increased by corticosteroids (candidate safety biomarkers). Proteins that showed significant drug-associated increases in DMD and IBD included insulin, angiotensinogen, afamin, leptin, growth hormone binding protein, and MMP3. Steroid-induced increases in insulin reflect the well-known induction of insulin resistance by corticosteroids. Insulin and glucose are typically measured in a fasting state, with fasting increased levels relatively specific and sensitive markers of insulin resistance and a hallmark phenotype of Type 2 diabetes. Here, insulin showed marked elevations in the non-fasted state, further supporting the profound induction of insulin resistance by corticosteroids. Two markers novel to this current report, increases of leptin (satiety hormone) and afamin (related to albumin), likely also reflect metabolic disturbances by corticosteroids, possibly downstream of insulin resistance. Serum afamin levels have recently been defined as a sensitive biomarker of metabolic syndrome^32^. Corticosteroids are also known to directly increase the expression of the angiotensinogen gene, and cause increased blood pressure through disruptions of the renin-angiotensin-aldosterone axis^33^. Our novel finding of increased serum angiotensinogen in DMD and IBD is likely a marker of these disturbances, and possibly a surrogate biomarker for salt-retention and Cushingoid appearance.

Increased growth hormone binding protein (GHBP) by corticosteroids is novel to this report, and is a candidate biomarker for stunting of growth in children caused by chronic corticosteroid treatment^34^,^35^. GHBP is the soluble proteolytic cleavage product of the membrane-bound human growth hormone receptor (GHR). The *GHR* gene is directly regulated by corticosteroids in a complex, dose-dependent manner^36^. However, serum levels of the soluble GHBP fragment of the receptor is regulated post-transcriptionally by metalloproteases that respond to inflammation and other cellular stimuli^37^,^38^. Soluble GHBP might compete for growth hormone binding, and thus is a negative regulator of growth hormone signaling. We hypothesize that increased concentrations of circulating GHBP compete with the membrane bound receptor for GH binding thus inhibiting its action on cells, leading to stunting of growth.

Drug-related increases in MMP3 showed the most dramatic changes of any biomarker in our study. MMP3 is a metalloproteinase involved in proteolytic degradation of the extracellular matrix, and has been studied as a joint and serum biomarker of tissue and cartilage damage in arthritis^39^,^40^,^41^. Progressive fibrosis is a feature of many chronic inflammatory states, including DMD and IBD. The significance of serum MMP3 for connective tissue remodeling and fibrosis in either DMD or IBD requires further study.

To employ serum biomarkers in drug development and optimization of therapies, individual biomarkers must be bridged to specific aspects of efficacy or safety. For example, to utilize the corticosteroid-responsive growth hormone binding protein (GHBP) as an acute, objective read-out of the safety concern of growth stunting, studies must show that serum GHBP levels are indeed predictive of later linear growth. For candidate pro-inflammatory efficacy biomarkers identified here, a specific marker that is changed after days of treatment (acutely responsive), and predicts later clinical benefit (surrogate outcome) could greatly speed drug development. A limitation of the current study is that we studied a relatively small number of children, and were not able to bridge specific biomarkers to specific aspects of safety or efficacy. However, we provide a panel of candidate biomarkers that may be studied in serum biobanks linked to clinical outcomes, and in prospective drug development studies. Towards this end, the biomarkers described in this report have been presented to both the FDA and EMA as exploratory biomarkers of efficacy and safety in the development of vamorolone, a potential replacement for corticosteroids^12^.

## Materials and Methods

### Serum sample collection from DMD patients and age matched healthy controls

Serum samples and clinical data from corticosteroid-treated and steroid-naïve DMD patients (male, 4–10 yrs), and age matched healthy controls were collected from participants enrolled in the Cooperative International Neuromuscular Research Group Duchenne Natural History Study (CINRG DNHS)^42^. The study protocol was approved by Institutional Review Board (IRB) at all participating institutions that provided us with DMD serum samples. These include Children’s National Health System, the University of California, Davis, CA, the University of Pittsburgh, Pittsburgh, PA and Alberta Children’s Hospital, Calgary, Canada. Informed consent was obtained from patients or their parent/legal guardian for biomarkers studies. All experiments were performed in accordance with approved guidelines and regulations. A standard operating procedure was sent to all participating sites to ensure consistent blood draw, serum sample preparation, storage and shipment. Demographics and characteristics of DMD participants are summarized in Table S1. GC-treated participants were typically prescribed either prednisone (0.75 mg/kg/day) or deflazacort (0.9 mg/kg/day). However, there was variation in physician practice regarding doses and dose schedules, as we have recently described in this same CINRG DNHS cohort^7^. Most DMD patients were also given dietary supplements such as vitamin D to bolster bone health, and nutraceuticals to bolster muscle health (Table S1).

Both cross-sectional and longitudinal subgroups were tested for corticosteroid-responsive serum biomarkers. The cross-sectional discovery group included 9 GC-naïve DMD patients, 5 GC-treated DMD patients, and 4 untreated healthy controls. The longitudinal validation group included 9 patients with DMD with samples collected before and after initiation of corticosteroid treatment. The average length of treatment was 4 months. There was no overlap in study participants between the cross-sectional and longitudinal groups.

### Serum sample collection from IBD patients

Eleven patients with IBD (2 ulcerative colitis, 9 Crohn’s disease, age 9–15 yrs) were enrolled into a biomarker protocol. Blood samples were taken at diagnosis prior to the initiation of GC, and then after corticosteroid treatment (paired samples for longitudinal analysis). Serum was prepared following the same SOP as for DMD patients. The average treatment time was 9 weeks. Patients were treated with standard dosing of 1 mg/kg/day prednisone once daily, up to a maximum of 40 mg daily. Demographics and characteristics of IBD patients are summarized in Table S2. Medication history above and beyond corticosteroids was noted (Table S2). This study was approved by the Children’s National Health System IRB and all experiments were performed in accordance with approved guidelines and regulations. All patients signed an informed consent for biomarkers study.

### Serum proteome profiling

Serum proteome profiling used 65 μL aliquots to perform a high throughput and highly multiplexed SOMAscan assay developed at Somalogic Inc. The principle of the SOMAscan assay has been described in more detail elsewhere^43^,^44^. Briefly, the SOMAscan assay is a panel of protein-specific Slow Off-rate Modified DNA aptamers (SOMAmers) that bind to the target protein with high affinity and specificity. In this study we used a SOMAscan version 3 consisting of 1,129 aptamers targeting 1,129 unique serum proteins. Each aptamer is tagged with short DNA sequence enabling high throughput quantification using custom hybridization array. Protein quantities are recorded as relative fluorescent units (RFU) from microarrays. All arrays were done using a dilution series of each sera sample so that the signal/noise ratio of each aptamer/protein pair was optimized (four dilutions).

### Statistical analysis

All protein expression levels were log transformed and normality of each verified by the Shapiro-Wilk normality test. Analyses were performed in DMD and IBD samples independently. DMD samples were segregated into two sample sets; a cross-sectional set consisting of participants having a single serum sample (treated or untreated DMD patients and unaffected, untreated age-matched controls), and a longitudinal set consisting of DMD boys with one sample prior to initiation of corticosteroids, and one or more samples after treatment. The two DMD sample sets were mutually exclusive so that no participant appeared in both. The first step of statistical analysis was a simple comparison of all 1,129 SOMAscan measured proteins between the cross-sectional DMD participants on corticosteroids and those steroid-naive. This analysis identified 45 proteins with a false discovery rate (FDR) adjusted p-value below the critical alpha level of 0.05. For the two validation cohorts (longitudinal DMD; longitudinal IBD), only those candidates defined by the cross-sectional analysis were analyzed.

The cross-sectional analysis was performed using linear regression models to compare mean protein expression levels between treated and untreated groups, while adjusting for age. The corticosteroid use coefficient and p-value, along with the adjusted means were derived for each group. Groups were also compared to untreated controls using the same methods, i.e. linear regression models with adjustment for age.

Longitudinal analyses were performed using mixed effects linear regression models with terms for corticosteroid use, time and age. These models offer several advantages. They allow the inclusion of different numbers of samples per individual and allow the corticosteroid use of each participant to vary (i.e. to start or stop corticosteroid use). Results are presented only for the corticosteroid use term, where the corticosteroid use coefficient represents the average expression level in participants while on corticosteroids as compared to those not on corticosteroids regardless of time. P-values for the longitudinal validation groups were not adjusted for multiple testing, as only a small number of tests were done using candidate markers from the discovery data set.

### Confirmation of SOMAscan data by ELISA assays

Insulin, leptin, C-C motif chemokine 22 (also known as macrophage-derived chemokine), and MMP3 were selected for confirmation of SOMAscan data using an electrochemiluminescence ELISA assay (Meso Scale Diagnostics, Rockville, MD). These four targets were selected based on the availability of well standardized and validated ELISA kits from Meso Scale Diagnostics. Serum aliquots and dilutions were prepared for each assay according to the recommendation of the manufacturer. Plates were analyzed using MSD MESO SECTOR S 600 plate reader and data recorded in ng/mL for each target. The relationship between SOMAscan and ELISA data was assessed using linear regression where SOMAscan values were the dependent variable. R squared and p-values are reported for each relationship.

### Steroid hormone profiling in serum samples by liquid chromatography tandem mass spectrometry

For steroid hormone profiling by LC-MS we followed a previously published protocol^21^. Briefly aliquots (250 μL) from each serum sample were spiked with a known amount of stable isotope labeled steroid hormone standards including aldosterone, progesterone, 17-hydroxyprogesterone, dehydroepiandrosterone, corticosterone, 11-deoxycortisol and testosterone and analyzed by reversed phase liquid chromatography coupled to a 4000 quadrupole mass spectrometer (ABISiex). Mass spectral data was processed and recorded for each steroid hormone in ng/mL.

## Additional Information

**How to cite this article**: Hathout, Y. *et al*. Serum pharmacodynamic biomarkers for chronic corticosteroid treatment of children. *Sci. Rep.* **6**, 31727; doi: 10.1038/srep31727 (2016).

## Supplementary Material

Supplementary Information

## Figures and Tables

**Table 1:** Steroidal pharmacodynamic biomarkers.

Protein name	DMD cross-sectional analysis				Untreated healthy volunteers		Longitudinal DMD p-value	Longitudinal IBD p-value
	CS naïve (n = 9)^a^	CS treated (n = 5)^a^	CS coefficient	p-value	CNT (n = 4)^a^	CS naïve DMD vs. CNT p-value		
Increased in DMD vs. control; decreased by corticosteroid in DMD								
CCL22	7.78 ± 0.07	7.01 ± 0.10	−0.77	<0.001	7.42 ± 0.13	0.025	<0.001	<0.001
IGFBP2	11.15 ± 0.12	10.42 ± 0.16	−0.73	0.005	10.50 ± 0.10	0.003	<0.001	0.028
C1R	4.87 ± 0.06	4.58 ± 0.08	−0.29	0.014	4.51 ± 0.05	0.005	ns	Ns
ITGa1/b1	7.57 ± 0.13	7.00 ± 0.18	−0.56	0.031	6.55 ± 0.09	0.002	0.026	Ns
FGG	10.38 ± 0.09	10.11 ± 0.13	−0.36	0.051	9.02 ± 0.03	<0.001	0.06	0.046
Similar in DMD vs. control; decreased by corticosteroid in DMD								
MMP12	7.98 ± 0.14	6.59 ± 0.2	−1.39	<0.001	7.89 ± 0.17	ns	<0.001	Ns
IL22RA2	8.81 ± 0.10	7.98 ± 0.13	−0.84	0.001	8.75 ± 0.18	ns	0.003	0.05
FCER2	9.12 ± 0.08	8.41 ± 0.11	−0.70	0.001	9.19 ± 0.03	ns	0.005	0.026
CCL21	9.59 ± 0.09	8.97 ± 0.13	−0.62	0.003	9.29 ± 0.14	ns	ns	Ns
LY9	8.61 ± 1.10	8.01 ± 0.14	−0.60	0.007	8.89 ± 0.07	ns	0.01	0.06
SLITRK5	8.81 ± 0.09	8.27 ± 0.12	−0.54	0.005	8.94 ± 0.16	ns	ns	Ns
LTa1/b2	6.15 ± 0.05	5.67 ± 0.07	−0.48	<0.001	6.12 ± 0.07	ns	0.003	ns
ANGPT2	6.60 ± 0.08	6.28 ± 0.11	−0.31	0.046	6.36 ± 0.10	ns	0.001	0.039
CSF1R	5.67 ± 0.06	5.27 ± 0.08	−0.29	0.013	5.80 ± 0.11	ns	ns	ns
Similar in DMD vs. control; increased by corticosteroid in DMD								
MMP3	5.81 ± 0.21	8.17 ± 0.28	2.36	<0.001	6.00 ± 0.02	ns	0.004	0.019
CNDP1	8.74 ± 0.16	9.42 ± 0.23	0.69	0.036	9.23 ± 0.10	0.06	ns	<0.001
AFM	9.98 ± 0.09	10.26 ± 0.13	0.37	0.041	10.29 ± 0.01	0.016	<0.001	0.006
AGT	7.23 ± 0.05	7.51 ± 0.07	0.29	0.009	7.58 ± 0.18	0.022	0.009	0.010
PROC	7.68 ± 0.04	7.95 ± 0.06	0.27	0.003	7.67 ± 0.08	ns	0.023	ns
INS	5.43 ± 0.05	5.90 ± 0.07	0.15	ns	5.57 ± 0.10	ns	0.032	<0.001
LEP	6.90 ± 0.09	7.05 ± 0.12	0.15	ns	6.92 ± 0.05	ns	0.002	ns
GHBP	6.20 ± 0.12	6.37 ± 0.17	0.07	ns	6.58 ± 0.09	0.033	0.026	0.002

^a^Value are log transformed and represent the mean and standard error for each protein in each sample set. DMD: Duchenne muscular dystrophy, IBD: inflammatory bowel disease, CNT: control, CS: corticosteroid.

**Table 2:** Corticosteroid pharmacodynamic biomarkers defined by longitudinal analysis of serum samples from corticosteroid naïve and corticosteroid treated DMD and IBD children.

Protein name	Pre and post corticosteroid treated DMD (n = 9)		Pre and post corticosteroid treated IBD (n = 11)		Description
	CS coefficient	p-value	CS coefficient	p-value	
Decreased by GC					
MMP12	−0.75	<0.001	−0.41	ns	Macrophage migration
IGFBP2	−0.65	<0.001	−0.54	0.03	Activated T cell proliferation
IL22RA2	−0.52	0.003	−0.67	0.05	T cell mediated inflammation
LTa1/b2	−0.44	0.003	−0.15	ns	B cell activation
CCL22	−0.37	<0.001	−1.04	<0.001	T cell migration
ANGPT2	−0.34	0.001	−0.41	0.04	Leukocyte migration
ITGa1/b1	−0.29	0.03	−0.12	ns	Neutrophil chemotaxis
FCER2	−0.27	0.005	−0.28	0.03	B cell differentiation
FGG	−0.24	ns	−0.71	0.05	Acute inflammation marker
LY9	−0.22	0.01	−0.32	0.06	T cell adhesion to other cells
Increased by GC					
MMP3	0.98	0.004	0.58	0.02	Extracellular matrix degradation
LEP	0.36	0.002	0.32	ns	Regulates body fat depot
AGT	0.34	0.009	0.35	0.01	Blood pressure regulator
INS	0.29	0.03	0.62	<0.001	Regulates glucose levels
GHBP	0.24	0.03	0.24	0.002	Regulate growth
AFM	0.23	<0.001	0.49	0.006	Involved in insulin resistance
PROC	0.11	0.023	0.06	ns	Anti-inflammatory/Cytoprotective

CS: corticosteroid.
