# Supplementary material for: Serum pharmacodynamic biomarkers for chronic corticosteroid treatment of children
Source: Sci Rep. 2016 Aug 17;6:31727. doi: 10.1038/srep31727 (PMC4987691; doi:10.1038/srep31727)
Supplement: Supplementary Information [file srep31727-s1.pdf]

Supplemental materials

Serum pharmacodynamic biomarkers for chronic corticosteroid treatment of children

Yetrib Hathout[1*], Laurie Conklin[1], Haeri Seol[1], Heather Gordish-Dressman[1], Kristy J Brown[1], Lauren P Morgenroth[1], Kanneboyina Nagaraju[1], Christopher R Heier[1], Jesse M Damsker[1], John N. van den Anker[1], Erik Henricson[2], Paula R Clemens[3], Jean K Mah[4], Craig McDonald[2] and Eric P Hoffman[1].

[1]Research Center for Genetic Medicine, Children's National Health Systems, Washington, DC 20010, USA.

[2]Department of Physical Medicine & Rehabilitation, University of California, Davis School of Medicine, Davis, CA 95618, USA

[3]Neurology Service, Department of Veterans Affairs Medical Center, Pittsburgh, PA and the Department of Neurology, University of Pittsburgh, Pittsburgh, PA

[4]Department of Pediatrics, Alberta Children's Hospital, Calgary, AB, Canada T3B 6A8

Supplemental Table S1: demographic of DMD patients and healthy controls used in this study

ID	Age	CS treatment	Length on CS (in weeks)	Additionnal medication
**Healthy control group**				
CNT	6	NA	NA	none
CNT	7.02	NA	NA	none
CNT	10.3	NA	NA	none
CNT	8.0	NA	NA	none
CNT	8.4	NA	NA	none
CNT	9.35	NA	NA	none
**Cross-sectional group**				
DMD-1 naïve	4.00	No	NA	vitamin D
DMD-2 naïve	4.30	No	NA	vitamin D
DMD-3 naïve	4.40	No	NA	vitamin D/Coenzyme Q10/Albuterol/Zyrtec
DMD-4 naïve	4.40	No	NA	Acetaminophen/Neurontin
DMD-5 naïve	4.90	No	NA	none
DMD-6 naïve	5.60	No	NA	none
DMD-7 naïve	6.80	No	NA	vitamin D/calcium
DMD-8 naïve	8.00	No	NA	Vitamin D/Calcium/Minerals
DMD-9 naïve	9.20	No	NA	Vitamin D/Calcium/Acetaminophen
DMD-10 + GC	4.30	Yes	6	multivitamins
DMD-11 + GC	5.60	Yes	12	vitamin D
DMD-12 + GC	7.40	Yes	12	vitamin D
DMD-13 + GC	9.50	Yes	18	vitamin D/calcium
DMD-14 + GC	9.60	Yes	18	vitamin D
**Longitudinal group**				
DMD patient 1	7.8	No	0	vitamin D
DMD patient 1	8.13	Yes	6	vitamin D/fish oil
DMD patient 1	8.38	Yes	9	vitamin D/fish oil
DMD patient 1	8.61	Yes	12	vitamin D/multivitamins
DMD patient 1	9.15	Yes	18	vitamin D/multiviatmins/ Albuterol/Idebenone
DMD patient 2	6.3	No	0	none
DMD patient 2	6.53	Yes	3	multivitamins
DMD patient 2	6.79	Yes	6	vitamin D/multivitamins

DMD patient 2	7.02	Yes	9	vitamin D/multivitamins
DMD patient 2	7.29	Yes	12	vitamin D/multivitamins
DMD patient 3	4.9	No	0	none
DMD patient 3	5.4	No	0	none
DMD patient 3	5.95	Yes	12	Acetaminophen
DMD patient 3	6.35	Yes	18	vitamin D/Non-steroidal anti-inflammatory medication
DMD patient 4	5.7	No	0	none
DMD patient 4	5.8	No	0	none
DMD patient 4	6.8	No	0	vitamin D
DMD patient 5	5.8	No	0	vitamin D/Amoxicillin
DMD patient 5	6.48	Yes	6	vitamin D
DMD patient 6	4.27	No	0	vitamin D/Calcium
DMD patient 6	4.30	No	0	vitamin D/Calcium
DMD patient 6	4.96	Yes	12	vitamin D
DMD patient 6	5.53	Yes	18	vitamin D
DMD patient 7	5.4	No	0	vitamin D/calcium
DMD patient 7	6.2	No	9	vitamin D/Calcium
DMD patient 7	6.92	Yes	12	vitamin D
DDM patient 8	5.0	No	0	vitamin D
DMD patient 8	5.81	Yes	12	vitamin D
DMD patient 8	6.25	Yes	18	vitamin D
DMD patient 9	7.7	No	0	vitamin D
DMD patient 9	8.36	Yes	9	vitamin D
DMD patient 9	8.74	Yes	12	vitamin D/Calcium

Supplemental Table S2: Demgraphic of pre and post glucocorticoids treated IBD patients

Patient ID	Age	Gender	Length on CS treatment (in weeks)	other medications
3	12	F	11	none
4	11	M	12	mesalamine
17	9	M	15	mesalamine, lansoprazole
26	9	M	5	mesalamine
27	10	M	6	mesalamine, lansoprazole
33	13	M	5	6MP, mesalamine, lansoprazole
54	12	M	10	mesalamine, lansoprazole
92	10	F	8	mesalamine
108	15	F	12	omeprazole, 6MP, mesalamine, propranolol, amytryptiline
21	15	F	3	mesalamine, lansoprazole
28	15	F	18	mesalamine, lansoprazole
